# Effect of the Application of *Ochrobactrum* sp.-Immobilised Biochar on the Remediation of Diesel-Contaminated Soil

**DOI:** 10.3390/toxics12040234

**Published:** 2024-03-22

**Authors:** Charles Chinyere Dike, Alka Rani Batra, Leadin S. Khudur, Kamrun Nahar, Andrew S. Ball

**Affiliations:** 1 ARC Training Centre for Transformation of Australia’s Biosolids Centre, RMIT University, Bundoora, Melbourne, VIC 3083, Australia; 2 School of Science, RMIT University, Bundoora, Melbourne, VIC 3083, Australia; 3 Environment Protection Authority Victoria, Centre for Applied Sciences, Ernest Jones Drive, Macleod, VIC 3085, Australia; 4 School of Engineering, RMIT University, Melbourne, VIC 3000, Australia

**Keywords:** bioaugmentation, biochar, biodegradation, bioremediation, hydrocarbon, petroleum, pyrochar, sewage sludge

## Abstract

The immobilisation of bacteria on biochar has shown potential for enhanced remediation of petroleum hydrocarbon-contaminated soil. However, there is a lack of knowledge regarding the effect of bacterial immobilisation on biosolids-derived biochar for the remediation of diesel-contaminated soil. This current study aimed to assess the impact of the immobilisation of an autochthonous hydrocarbonoclastic bacteria, *Ochrobacterium* sp. (BIB) on biosolids-derived biochar for the remediation of diesel-contaminated soil. Additionally, the effect of fertiliser application on the efficacy of the BIB treatment was investigated. Biochar (BC) application alone led to significantly higher hydrocarbon removal than the control treatment at all sampling times (4887–11,589 mg/kg higher). When *Ochrobacterium* sp. was immobilised on biochar (BIB), the hydrocarbon removal was greater than BC by 5533 mg/kg and 1607 mg/kg at weeks 10 and 22, respectively. However, when BIB was co-applied with fertiliser (BIBF), hydrocarbon removal was lower than BIB alone by 6987–11,767 mg/kg. Quantitative PCR (q-PCR) analysis revealed that the gene related to *Ochrobacterium* sp. was higher in BIB than in the BC treatment, which likely contributed to higher hydrocarbon removal in the BIB treatment. The results of the q-PCR analysis for the presence of *alkB* genes and FTIR analysis suggest that the degradation of alkane contributed to hydrocarbon removal. The findings of this study demonstrate that bacterial immobilisation on biosolids-derived biochar is a promising technique for the remediation of diesel-contaminated soil. Future studies should focus on optimising the immobilisation process for enhanced hydrocarbon removal.

## 1. Introduction

Crude oil is an important natural resource for energy generation and the production of raw materials for the industry. The 2% average per annual increase in global oil consumption between 1965 and 2020 attests to how relevant oil remains to the world [1]. Nonetheless, petroleum/crude oil leakage and accidental release into the environment frequently occur during the exploration, refining, transportation, and storage of petroleum/crude oil and its derived products [2]. Soil pollution by petroleum hydrocarbons is a common problem in many countries [3,4]. For example, in Nigeria, between 2006 and June 2022, 4102 crude and refined oil spills on land were reported in the country’s oil spill monitor [4]. Diesel is a product of crude oil, a frequent progenitor for petroleum hydrocarbon pollution in the environment [5]. Acute and chronic effects on humans and plants occur after exposure to diesel oil [6]. For example, Bona et al. [7] reported that the seedling growth of *Schinus terebinthifolius* was significantly affected by exposure to diesel.

Remediation of crude oil-contaminated soils is necessary due to the detrimental effects of exposure to this contaminant [8]. Although several remediation techniques are available for oil/petroleum hydrocarbon-impacted environments [9], remediation of contaminated soil remains a concern. This is owing to the shortcomings associated with existing remediation techniques. For example, thermal desorption is expensive and prone to secondary pollution [10]. Recently, biochar has gained relevance in the remediation of hydrocarbon-contaminated soil. Biochar is a carbon-based product obtained from the thermochemical decomposition of biomass, including waste materials, in an oxygen-limited environment [11]. The application of biochar to contaminated soil has been shown to enhance hydrocarbon removal by up to 2.1 times [12,13,14]. For example, Aziz, Ali, Farooq, Jamal, Liu, He, Guo, Urynowicz and Huang [12] showed that adding sludge-derived biochar led to a minimum of 101% greater removal than the control treatment. Biodegradation is one of the ways through which biochar is thought to accelerate the remediation of hydrocarbon-contaminated soil [12]. In biodegradation, the contaminant is broken down to a harmless product at the end of mineralisation rather than being transferred from the contaminant site. The biodegradation of petroleum hydrocarbons is affected by the microbial community present [15]. It is possible that contaminated soil may not have the right autochthonous hydrocarbon-degrading microbial community [16]. The above scenario could impede the biodegradation effect of biochar on hydrocarbon removal. The co-application of biochar with bioaugmentation, including microbes, has been advocated in the remediation of petroleum hydrocarbon-contaminated soil [17]. Bioaugmentation can involve the use of autochthonous, allochthonous, or genetically engineered microbes [18], with the autochthonous form being the cheapest and least stressful to use from a regulatory and commercial perspective. Evidence in published literature confirms that biochar’s co-application with bioaugmentation (microbe) leads to increased hydrocarbon removal relative to biochar treatment on its own [19,20,21,22].

The co-application of biochar with microbes has been conducted using immobilised, free-living, or other associations [19,20,23]. Compared to free-living co-application, the immobilised form is more effective in remediating hydrocarbon-contaminated soil [24,25]. This is likely because the advantage of immobilising bacteria to biochar may not be realised with free-living bacterial addition [17]. There have been attempts to investigate the effect of bacteria immobilised biochar (BIB) on the remediation of petroleum hydrocarbon-contaminated soil [19,21,25,26,27,28]. Guo et al. [19] found that irrespective of the wheat bran biochar pyrolysis temperature, the immobilisation of biochar with bacteria consortia resulted in statistically significant hydrocarbon removal, compared to the corresponding biochar treatment. A most recent study observed that the immobilisation of biochar with *Shewanella putrefaciens* led to 42% higher removal than the biochar treatment alone after 60 days [22]. Galitskaya et al. [26] showed that immobilising *Pseudomonas aeruginosa* or *Acinetobacter radioresistens* on birch waste biochar did not result in a statistically significant difference in hydrocarbon removal, relative to the biochar treatment.

Despite these attempts targeted at investigating the effect of BIB, there is a lack of knowledge regarding the role of BIB in the remediation of diesel-contaminated soil when biochar is produced from biosolids. Using biosolids-derived biochar in bacterial immobilisation offers an alternative approach for managing problematic waste. The rapid rise in the amount of biosolids generated makes sustainable biosolids management a priority issue in our contemporary world [29]. Considering that the effect of bacteria immobilised biochar (BIB) on hydrocarbon removal has been examined in the absence [19,21,22,26] and presence [24,25] of supplementary nitrogen and phosphate, it is important to see whether fertiliser addition would be beneficial for BIB in the remediation of diesel-contaminated soil when biosolids-derived biochar is used for bacterial immobilisation. Since the goal of adding nutrients to hydrocarbon-contaminated soils is to compensate for the alteration in the carbon-to-nitrogen (C/N) ratio induced by the hydrocarbon, a soil with a high C/N ratio (27) was used for this study.

The chemical structure or functional group of compounds can be used to group petroleum hydrocarbons [30]. The RemScan technology, an alternative to gas chromatography/mass spectrometry (GC/MS) analysis with benefits in terms of cost and speed, provides an idea of the concentration of the total C_10_–C_40_ present in the soil [31,32]. However, it does not give an idea of the changes in the functional groups or chemical structure of the hydrocarbon with time or treatments. With the integration of Fourier transform infrared (FTIR) spectroscopy in remediation, it is possible to decipher changes in the functional groups or chemical structure. Fourier transform infrared spectroscopy has been widely applied to study changes in functional groups of contaminants and can be rapidly used to characterise different functional groups, including aliphatic and aromatic [30]. This understanding can offer insight into the influence of time and treatment on hydrocarbon fraction. Therefore, the RemScan was used to study the total petroleum hydrocarbon (TPH) in this study, while FTIR was utilised for the assessment of functional groups.

This current study is unique because it is the first study to investigate the role of bacteria immobilised biochar in the remediation of diesel-contaminated soil when biochar has been produced from biosolids. In addition, the study is also the first to assess the effect of *Ochrobacterium* immobilised biochar on the remediation of Australian soil contaminated with diesel. The specific objectives were to: (i) isolate bacteria from hydrocarbon-contaminated soil and compare their hydrocarbon degradation efficiency; (ii) evaluate the effect of bacteria immobilised biochar on the remediation of diesel-contaminated soil; (iii) assess the role of fertiliser on the efficacy of bacteria immobilised biochar on hydrocarbon removal; (iv) assess the abundance of genes related to alkane degrading bacteria (*alkB*), *Ochrobactrum* sp. (OCB), and encoding for the total bacterial population (16S rRNA); and (v) assess the degradation of the diesel using FTIR spectroscopy.

## 2. Materials and Methods

### 2.1. Soil and Biochar

Pristine soil was obtained from Whittlesea, Melbourne, Victoria, and had a pH of 7.6, total carbon of 2.23%, total nitrogen of 0.22%, and total phosphorus content of 313 mg/kg [33]. The soil used for bacteria isolation was the same Whittlesea soil as above with diesel, biochar, and sodium azide (BN) (unpublished).

The biochar used was produced from biosolids obtained from the Mount Martha Water Recycling plant (38°16′06″ S and 145°03′31″ E) operated by South East Water Corporation, Melbourne, Australia. In this plant, the source of biosolids was a municipal wastewater treatment plant that receives domestic wastewater. Approximately 13 million tonnes of sewage are treated in this treatment plant. Sewage is treated and converted to biosolids via various processes, including activated processes, anaerobic digestion, dewatering, and solar drying, before being stockpiled.

Before pyrolysis, the biosolids were dried in the incubator for over 18 h, transferred into a crucible, and pyrolysed in a muffle furnace at 900 °C for 3 h at a heating rate of 10 °C/min. The produced biochar was passed through a 1 mm mesh. The biochar had a volatile matter of 3.15 ± 0.21%, fixed carbon of 20.18 ± 5.26%, and ash content of 76.26 ± 4.79%.

### 2.2. Isolation of Bacteria from Diesel-Contaminated Soil

Bacteria were isolated from soil BN as described in Section 2.1. The methods previously described in the literature with modifications were used for bacterial immobilisation [19,34,35]. Briefly, soil (2.5 g) was added to minimal salt media (MSM, KH_2_PO_4_ 15 g/L, NaCl 2.5 g/L, Na_2_HPO_4_ 33.9 g/L, and NH_4_Cl 5 g/L, 25 mL) with diesel at a concentration of 10 mL/L and incubated for 7 days at 30 °C at 150 rpm for the first cycle. Following incubation, an aliquot (2.5 mL) from the previous cycle of incubation was transferred to fresh MSM (22.5 mL) with a higher diesel concentration and incubated at 30 °C at 150 rpm for 5 d. This was repeated three times, with an increment in the diesel concentration at each cycle; the range of concentration of diesel in the fresh MSM culture after the first cycle was around 200–800 mL/L.

From the final MSM culture, serial dilution of the culture was carried out. An aliquot (100 μL) was spread on Lueri Bertani broth (LB) with agar, Lennox (Sigma-Aldrich, Massachusetts, US). Plates were incubated at 30 °C for 7 d. Colonies were streaked onto LB agar plates and isolates purified. Three distinct colonies were isolated after a series of isolations, namely, isolates A, B, and C.

### 2.3. Identification of Bacteria Isolates

The three (3) bacterial isolates were identified using Matrix assisted laser desorption ionisation time of flight mass spectrometry (MALDI-TOF MS). This method of bacterial identification involves mass spectrometry and was carried out in a MALDI-TOF MS device (Bruker Corporation, MA, Billerica, USA) using Flex control software (version 3.4, build 135.10) [36]. The database used to identify strains was the MBT Compass Library revision K, version 12 (2022). The MALDI biotyper utilises a proteomics technique and acquires a bacterial protein mass list for identification. Briefly, a toothpick was used to pick bacterial samples from a culture plate to a spot on the target plate. The bacterium was smeared on the spots on the target plate. This was followed by adding 70% formic acid (1 μL), mixing thoroughly with a toothpick, and letting it dry completely. An aliquot (1 μL) of the matrix was later added to the dry sample and left to dry. The target plate containing the dried sample was placed on the dock of the equipment and was read.

Further identification was carried out for isolate C using 16S rRNA Sanger sequencing. Colonies of the bacteria isolates were suspended in PrepMan buffer (100 μL) and were sent to the Australian Genome Research Facility Ltd (AGRF) in Melbourne, Victoria, Australia, for Sanger sequencing. The database that was used for the Sanger sequencing was Greengenes v13_8, while Blastn with default settings was used to do the search.

### 2.4. Assessment of the Efficacy of Bacterial Isolates to Remediate Diesel-Contaminated Soil

The three isolated bacteria (A, B, and C) were examined for their hydrocarbon-degrading efficiency by introducing them individually to diesel-contaminated soil, with a TPH concentration of 32,400 ± 937 mg/kg. For each of the bacterial isolates, colonies from the streak plate were cultured in LB broth for 19 h at 150 rpm and 30 °C to attain an optical density of 0.8–2 at 600 nm. To ensure that an equal optical density of 1.09 at 600 nm was used for the three bacteria, the volume was normalised. The cells were washed in 0.9% NaCl and resuspended in 0.9% NaCl after washing. An equal volume of bacterial suspension A, B, and C was added to separate pots containing diesel-contaminated soil, in triplicate. A non-amended control (without bacteria) treatment was included, and all pots were incubated at room temperature in the laboratory, with sampling on days 7, 14, and 37 to determine the hydrocarbon concentration.

### 2.5. Bacterial Immobilisation on Biochar

The bacterium exhibiting the greatest efficacy from the preliminary experiment in Section 2.4 was chosen for immobilisation on biochar (isolate C). The bacteria were streaked on an LB agar and incubated at 30 °C for at least 24 h. Distinct colonies were transferred to LB broth and cultured at 30 °C with shaking (150 rpm) for 19 h. The bacteria were centrifuged at 5000 rpm for 10 min (Heraeus Megafuge 8R, Thermo Scientific, Waltham, MA, USA), then rinsed with 0.9% NaCl three to four times. The bacterial pellets were resuspended in sterile 0.9% NaCl. A heterotrophic bacterial count was assessed by dilution on LB agar, incubated for more than 24 h at 30 °C. The heterotrophic bacterial count after culturing was 6.5 × 10^9^ ± 1.4 × 10^8^ CFU/mL.

A 1:5 (*w*/*v*) ratio was used to immobilise the bacterium to the biochar [21]. Biochar (6 g) and 30 mL bacterial suspension (6.5 × 10^9^ CFU/mL) were transferred to a 50 mL centrifuge tube incubated for 24 h at 30 °C and 150 rpm. The mixture was centrifuged at 1000 rpm for 20 min, followed by another 5 min centrifugation at 1000 rpm (Heraeus Megafuge 8R, Thermo Scientific, Waltham, MA, USA). The resultant pellet was washed with 0.9% NaCl three times and centrifuged at 1000 rpm for 20 min (Heraeus Megafuge 8R, Thermo Scientific, Waltham, MA, USA). The immobilised biochar was dried in a biosafety cabinet for 6 d at room temperature, followed by drying at 30 °C for 4 d in the same cabinet.

### 2.6. Bioremediation Mesocosm Experiment

Pristine soil (sieved using a 4 mm sieve) was contaminated with diesel at 6.4% (*v*/*w*), then mixed and left in the fume hood for 24 h. The soil was mixed, and 180 g was dispensed into 30 glass containers, then placed inside a plastic mesocosm of equal diameter. The appropriate treatments were added to different mesocosms in triplicate, as described in Table 1. For the bacteria only treatment, bacteria (45 mL) were centrifuged (Heraeus Megafuge 8R, Thermo Scientific, Waltham, MA, USA) and resuspended in 5 mL of 0.9% NaCl. Sodium chloride (5 mL, 0.9%) was added to the mesocosms for all other treatments. Water (1.5–4.2 mL) was added at least once every two weeks for the first five (5) weeks. From week 6, the moisture content was regulated to 11–18% once every week by adding water when necessary. The moisture content was measured by drying the soil sample in an oven for at least 12 h at 105 °C. The weight of the pot was measured weekly, and water was added when necessary to achieve the required moisture. The soil was mixed at least once every week and sampled in weeks 3, 6, 10, 14, 18 and 22.

### 2.7. Total Petroleum Hydrocarbon (TPH) Analysis

The TPH concentration was assessed using RemScan Technology (Ziltek, South Australia, Australia) [32]. The device utilises a diffuse reflectance (mid)-infrared Fourier transform (DRIFT) spectrometer for assessment of TPH [32]. The amount of soil sampled for RemScan analysis was >20 g. Before the measurement of TPH, the soil samples were air-dried for 24 h in the fume hood, ground in most cases (except week 0 and 3) and passed through a 2 mm sieve.

### 2.8. Molecular Microbiological Analysis

#### 2.8.1. Isolation of DNA from Soil and Bacteria Samples

DNA was isolated from the soil, bacteria, biochar, and bacteria immobilised biochar samples using a Power Soil Kit (Qiagen, Hilden, Germany). For the bacterial isolate, a sterile loop was used to transfer the bacterial isolates to the power beads tube. A sample weight of 0.25 g was used for the soil, biochar, and bacterial immobilised biochar. DNA extraction was conducted using the manufacturer’s instructions. The DNA isolated from the bacteria had a quality (A_260_/A_280_) of 1.87–1.88 and concentration of 68.7–69.7 ng/μL.

#### 2.8.2. Quantitative PCR (qPCR) Analysis

Real-time PCR was carried out to quantify the number of copies of: (i) *alkB* gene encoding alkane degrading bacteria, (ii) 16S rRNA encoding for the total bacteria population, and (iii) 16S rRNA that encodes a gene related to *Ochrobactrum*, using a Qiagen Rotor Gene machine (Qiagen, Germantown, MD, USA). The genes were referred to as 16S rRNA and OCB. qPCR analysis of DNA extracted from the biochar, soil, and bacteria immobilised biochar were all conducted in replicates. A 20 μL reaction was used for amplification of the two genes assessed, which comprised 0.4 μL forward primer (10 pmol/μL), 0.4 μL reverse primer (10 pmol/μL), 8.2 μL nuclease free water, 10 μL SensiFAST SYBR^®^ No-ROX Kit master mix (Meridian Bioscience, Newtown, OH, USA), and 1 μL DNA sample [37]. The primer set used for the *alkB* gene comprised *alkB*-F (5′-AAYACIGCICAYGARCTIGGICAYAA-3′) and *alkB*-R (5′-GCRTGRTGRTCIGARTGICGYTG-3′) [38]. The primers used for the *16S rRNA* gene were 341-F (5′CCT ACGGGAGGCAGCAG3′) and 518-R (5′-ATTACCGCGGCTGCTGG-3′) [39], while 5′-CTACCAAGGCGACGATCCAT-3′ and 5′-GGGGCTTCTTCTCCGGTTAC-3′ were used for the OCB gene as forward and reverse primers, respectively. The primer for the *OCB* gene was obtained from the National Centre for Biotechnology Information website, with an ascension number DM110786.1. Before the use of this primer, PCR and gel electrophoresis was carried out using the bacteria DNA. Details are provided in Text S1.

The cycling conditions used for the *alkB* gene were: (i) initial denaturation at 95 °C (10 min); (ii) 5 cycles at 95 °C (45 s); (iii) 1 min at 62 °C; (iv) 45 s at 72 °C; (v) 45 s at 95 °C (40 cycles); (vi) 1 min at 57 °C and 45 s at 72 °C [37,38]. When each cycle was completed, data was retrieved at 78 °C and for the analysis of the melting curve, a final cycle was carried out comprising 95 °C (15 s), 60 °C (30 s), and 95 °C cycles (15 s each) [38]. The *16S rRNA* and *OCB* genes were amplified using the same cycling conditions. The conditions: (i) initial denaturation step at 95 °C for 5 min; (ii) 40 cycles at 95 °C denaturation for 10 s; (iii) annealing at 55 °C for 30 s; (iv) 72 °C extension for 30 s; and (v) 80 °C primer dimer removal and signal acquisition (10 s) [40]. A standard curve for each gene was created using serial dilutions of the cleaned PCR products of the gene [40]. A plot of the cycle threshold (CT) values from the serial dilutions versus the log of their original copy number was prepared, and then a standard curve was produced using linear regression [37]. To calculate the copies of each gene, the CT value was correlated with the standard curve of the gene of interest and reported as log_10_ gene copy number/g dry soil [41].

### 2.9. Fourier Transform Infrared (FTIR) Analysis of the Soil

For FTIR analysis, soil samples were dried for at least 12 h in the fume hood. FTIR identified functional groups of soils by scanning 650–4000 cm^−1^ with 16 scanning times at 4 cm^−1^ resolutions using Frontier FTIR Spectroscopy (Spectrum 100, Perkin Elmer, Waltham, MA, USA). The spectra were reported in an absorbance mode.

### 2.10. Statistical and Kinetic Analysis

All soil analyses were carried out in replicates. Results were expressed as the mean of the replicates and the standard deviation. One-way analysis of variance (ANOVA) was used to assess the statistical difference at *p* < 0.05 using Minitab version 21.1 software (Minitab, State College, PA, USA). Microsoft Excel Version 2401 (Microsoft, Washington, DC, USA) was used to plot the kinetic curves of the different treatments.

First-order kinetics was used for the kinetics of bioremediation in this study [42]. The equation of the first-order kinetics is as follows:

C_t_ = C_0_·exp(−kt) (1)

where C_t_ is the concentration of the contaminant at the time t (mg/kg), k is the first-order kinetic constant (day^−1^), C_0_ is the concentration at the beginning (mg/kg), and t is the time (days) [42]. The half-life (DT_50_) of biodegradation was calculated using Equation (2):

(2) $${\mathrm{DT}}_{50}=ln2/k$$

where k represents the rate constant (day^−1^) [43].

## 3. Results and Discussion

### 3.1. Isolation, Identification, and Screening of Autochthonous Hydrocarbonoclastic Bacteria

Three distinct bacteria were isolated from the diesel-contaminated soil (isolates A, B, and C). MALDI-TOF MS examination identified isolates A, B, and C as *Achromobacter denitrificans*, *Stenotrophomonas* sp., and *Ochrobactrum grignonense*, respectively. The confidence scores were 1.85, 2.14, and 1.89 for identification of isolates A, B, and C, respectively. Isolate C was further identified using Sanger Sequencing with 100% identity as *Ochrobactrum* sp. Bacteria belonging to the Achromobacter, Stenotrophomonas, and Ochrobactrum genus have previously been isolated from soils contaminated with petroleum hydrocarbon [44,45,46,47].

**Figure 1:**
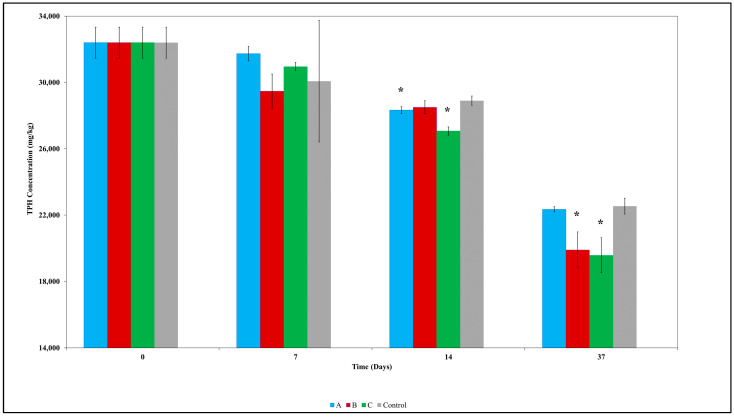
Total petroleum hydrocarbon (TPH) removal over 37 days in contaminated soils bioaugmented with bacterial isolates and the control (unamended soil). Values are the mean of triplicate measurements, while the error bar represents the standard deviation of the mean. Asterisk (*) denotes a significant difference (*p* < 0.05) between the control and any other treatment (A, B, and C) at the same sampling time. A: *Achromobacter denitrificans*; B: *Stenotrophomonas* sp.; C: *Ochrobactrum* sp.; Control: No bacterial amendment.

The isolates were examined for their ability to remediate diesel-contaminated soil through bioaugmentation (Figure 1). Initially, at day 7, all bacterial amended treatments showed similar TPH concentration profiles compared to the control (*p* < 0.05). However, by day 14, TPH concentrations were significantly lower in soils with isolates A and C. At the end of incubation, only treatments amended with isolates B and C had a significantly lower hydrocarbon concentration (*p* < 0.05) compared to the control (Figure 1). Wu et al. [48] observed that bioaugmentation only showed a significant difference from the control from week 2. Comparing treatment with isolates B and C, soils amended with isolate C showed the lowest average and standard deviation and, on this basis, was selected for biochar immobilisation and subsequent incubation study.

### 3.2. Immobilisation of Bacteria on Biochar

To assess whether the immobilisation of the bacteria on the biochar was successful, quantitative PCR and proximate analysis were carried out on the biochar before and after immobilisation. The number of the 16S rRNA gene copies increased by 1.1 log_10_ gene copies/g following immobilisation on the biochar, confirming the adsorption of bacteria on the biochar. Further, proximate analysis of the biochar showed an increase in both the volatile matter and fixed carbon after bacterial immobilisation on the biochar, while the ash content decreased after immobilisation (Table S1).

### 3.3. Remediation of Contaminated Soil

#### 3.3.1. Impact of Biochar on Remediation of Contaminated Soil

**Figure 2:**
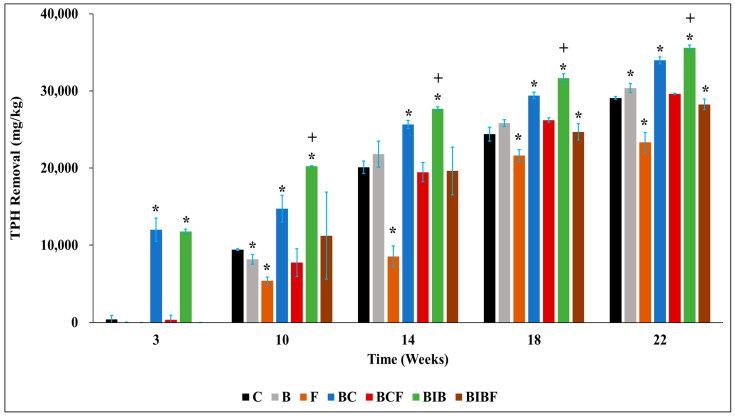
TPH removal in the different treatments over a 22-week incubation. Values represent the mean of replicate measurements, while the error bar represents the standard deviation of the mean. The hydrocarbon concentration at the beginning was 62,027 ± 1209 mg/kg. C: Control (unamended soil); B: Bacteria alone added; F: 2% fertiliser alone added; BC: 5% *w*/*w* biochar; BCF: 5% *w*/*w* biochar + 2% fertiliser; BIB: Bacteria immobilised biochar; BIBF: Bacteria immobilised biochar + 2% fertiliser. Plus (+) shows that a significant difference (*p* < 0.05) exists between BC and BIB at the same time, while asterisks (*) shows that C differs significantly (*p* < 0.05) from other treatments.

To assess the efficacy of adding biochar to hydrocarbon-contaminated soils (6.2% total petroleum hydrocarbon concentration), TPH concentration was monitored over 22 weeks across different treatments (Figure 2). The results showed that the hydrocarbon concentration decreased in all the biochar treatments and the control at the end of incubation (Figure 2), which is consistent with the literature [12,13,49]. At week 3, negligible removal occurred in the control treatment; however, a significant reduction of 11,993 mg/kg occurred in the biochar treatment. The hydrocarbon removal in the biochar treatment remained higher (*p* < 0.05) than the control throughout the incubation, with 17% greater removal at week 22 (Figure 2). The increased difference in hydrocarbon removal between both treatments in the early stages of remediation in the biochar treatment was consistent with our previous work involving biosolids biochar [13]. The fate of the components of crude oil in terms of degradation varies, with n-alkanes being easier to degrade compared to the branched alkanes [50]. It is possible that the readily degradable fraction of the hydrocarbon was consumed faster by the microbes in the biochar treatment [51], resulting in a reduction in the rate of remediation in later stages. It is also possible that some metabolites produced during biodegradation inhibit microbial metabolism and, subsequently, hydrocarbon removal [52]. Biochar can serve as a biostimulating agent and thus enhance hydrocarbon removal because of its ability to support soil microbial communities by providing habitat or improving soil [53]. Our results on the effectiveness of biochar in enhancing hydrocarbon removal agree with other reports in the literature [12,13,49,54].

#### 3.3.2. Impact of Bacteria Immobilised Biochar on the Remediation of Contaminated Soil

Bacteria were immobilised on biochar to improve the efficacy of biochar in the remediation of diesel-contaminated soil. Figure 2 indicates that there was no significant difference (*p* < 0.05) in hydrocarbon removal in soils amended with either bacteria immobilised biochar (BIB) or biochar (BC) at week 3. However, at 10 weeks, BIB was more effective in hydrocarbon removal than BC, achieving a significantly greater reduction of 5533 mg/kg than the BC treatment (*p* < 0.05). A recent study found a greater differences between both treatments in terms of residual hydrocarbon concentration from day 20 [22]. After week 10 in our current study, BIB remained significantly higher (*p* < 0.05) than the biochar treatment until the end of the incubation (week 22). It is likely that BIB was more effective in hydrocarbon removal than the BC treatment because of the co-application of biochar with *Ochrobactrum* sp. Many strains of this bacteria degrade a range of contaminants such as polycyclic aromatic hydrocarbons, herbicides, crude oil, and phenols [55]. Xu et al. [56] reported that the bioaugmentation of *Ochrobactrum* sp. resulted in higher PAH removal than the control treatment. However, adding the bacteria alone was largely non-beneficial, as this treatment did not result in significantly greater removal (*p* < 0.05) relative to the control, except at week 22 (Figure 2). Therefore, greater TPH removal in the BIB-amended soil could be due to the advantages of combining both remediation techniques (biochar and bioaugmentation); a proposed mechanism has been summarised in a previous review (Figure S1) [17]. Briefly, biochar enhances the mass transfer of the contaminant from the soil to the immobilised biochar, reducing the contact distance between the immobilised bacteria and the contaminant [17]. The enhanced TPH removal observed in BIB is consistent with reports from previous works [19,21,22].

Interestingly, the addition of fertiliser to contaminated soil along with the BIB resulted in a significant reduction (*p* < 0.05) in TPH removal compared with the BIB treatment, except at week 10 (Figure 2), implying that the fertiliser was non-beneficial; further when fertiliser alone was added to contaminated soil, a lower TPH removal was observed relative to the untreated soil (control) (Figure 2). To the best of our knowledge, there have been a lack of studies comparing the effect of fertiliser addition on the efficacy of BIB involving biosolids biochar. There is a possibility that the 2% fertiliser addition may have led to a low soil C/N ratio, which further resulted in the slowdown of hydrocarbon removal. This assertion is supported by the F and BCF treatment results, which showed non-beneficial effect on hydrocarbon removal compared to the control and BC treatment, respectively (Figure 2). This observation stresses the need for care in the application of supplementary fertilisers.

#### 3.3.3. Remediation Kinetics and Prediction

In line with the Environmental Protection Authority (EPA) Victoria Priority waste category classification [57], none of the treatments met the threshold for fill material requirement after 22 weeks of incubation (1000 mg/kg). However, treatment BIB and BC achieved the maximum level for category B (40,000 mg/kg) at week 14, while others, including the control, did not until week 18 or 22 [57]. Remediation kinetics was used to predict when the treatments would achieve the EPA Victoria soil threshold. This can be used to determine the concentration of the contaminants at any given time, which can determine when a threshold would be achieved [58]. The kinetics equation, R^2^, and half-life are shown in Table S2, and the R^2^ of the different treatments varied between 0.84–0.99, which denotes that almost all the treatments fitted well with first order kinetics. Table S2 demonstrates that it would take BIB 131 days for half of the contaminants to be degraded, whereas the BC and C treatments would achieve that in 141 and 157 days, respectively. Predictions made with the kinetics showed that the EPA Victoria material threshold would be achieved in the control 23 and 14 weeks after BIB and BC treatments meet the threshold, respectively (Table S3). Based on predictions from the kinetics, the addition of biochar or BIB would enable remediators to meet the threshold faster than when soils are remediated unaided.

### 3.4. FTIR Analysis of the Soil

The changes in the chemical structure of the petroleum hydrocarbons in the soil as a function of time and treatment were assessed using FTIR. The changes in the functional groups or chemical structure before and after remediation can provide an insight into the degradation of contaminants [59]. The intensity of peaks (2853, 2923, and 2953 cm^−1^), associated with -CH_3_ and -CH_2_ in aliphatic compounds decreased at week 22 in all treatments (Table S4). This confirmed that the degradation of aliphatic compounds contributed to a reduction in the hydrocarbon concentration in the soil. A comparison of weeks 10 and 22, showed that the differences in the intensity of these peaks among these treatments were wider at week 10 than week 22 (Table S4). This corroborates the results of the TPH removal, where greater differences in hydrocarbon removal among the three different treatments (C, BC, and BIB) were observed at week 10 compared to week 22 (Figure 2). These results indicate that the lack of difference in hydrocarbon removal among the treatments at week 22 was because most of the aliphatic compounds (easily degradable fraction) were degraded at this time, and thus most of the remaining hydrocarbon required more time to degrade.

### 3.5. Quantification of Functional Genes

**Figure 3:**
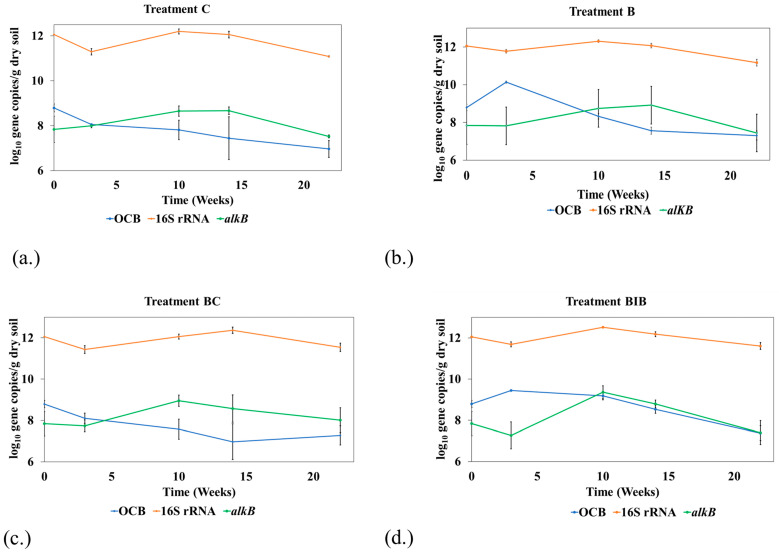
Number of copies of OCB (log_10_ gene copies/g dry soil), 16S rRNA (log_10_ gene copies/g dry soil), and *alkB* gene (log_10_ gene copies/g dry soil) in (**a**) C; (**b**) B; (**c**) BC; and (**d**) BIB over 22 weeks. Values represent the mean of triplicate measurements, except treatment BC at week 14 (duplicate readings only). The errors bar represents the standard deviation of the mean. C: Control (unamended soil); B: Bacteria alone added to soil; BC: 5% *w*/*w* biochar; BIB: Bacteria immobilised biochar.

Quantitative PCR was carried out on a 16S rRNA gene related to *Ochrobactrum* sp. (OCB) to understand the fate of the introduced autochthonous bacteria in the soil and to see if co-applying biochar with the bacteria contributed to differences in hydrocarbon removal between BC and BIB. At week 3, the copy number of this gene was higher in all treatments with the addition of the bacteria (B and BIB), confirming the success of the bioaugmentation. These findings are consistent with previous work where the copies of a gene targeting Mycobacterium *nidA* were higher in treatment with bioaugmentation at day 0 and 18 than those without bioaugmentation [27]. Interestingly, in soils amended with *Ochrobactrum* sp., the number of gene copies initially increased but then decreased over time (Figure 3b,d); in soils without bacterial amendment, copy numbers declined over time (Figure 3a,c). At week 3, this gene had a higher abundance in treatment B than BIB (*p* < 0.05). Partial immobilisation of the bacteria to the biochar and the loss of the cells during the BIB preparation process may be responsible for the lower abundance in BIB at this time [27]. However, the copy number of this gene was significantly higher (*p* < 0.05) in BIB than in B subsequently, except at week 22, suggesting the benefit of immobilising the introduced microorganism (Figure 3b,d).

Overall, the numbers of OCB genes were significantly higher (*p* < 0.05) in BIB than in the BC treatment at all sampling times, with exception of week 22. Previous studies found that the abundance of the introduced bacteria was higher in the biochar immobilised treatment than in the biochar treatment [21,27]. For example, Song et al. [21] observed that the relative abundances of the Sphingomonas genus was significantly higher in the immobilised treatment than the biochar treatment (*p* < 0.05). Although the gene targeted in this our current study was not only specific to Ochrobactrum, it is likely that the higher abundance of this gene may be due to the presence of *Ochrobactrum* sp. in the BIB treatments. This could have contributed to higher hydrocarbon removal observed in BIB treatments compared to BC. Ochrobactrum strains are reported to degrade a range of contaminants, including crude oil [55]. For example, increased PAH removal was observed in a previous study amended with *Ochrobactrum* sp. relative to the control [56].

Bioaugmentation of soil with a hydrocarbon-degrading bacterium in a protective environment could give the BIB an increased advantage over the BC treatment, considering that the indigenous bacteria may not function to their full potential because of the resultant effect of the toxic environment and competition.

The gene encoding the total bacteria population (16S rRNA) was also quantified to understand the effect of remediation time and treatments on the bacteria population (Figure 3). The abundance of this gene in the various treatments ranged from 11.08 log_10_ to 12.5 log_10_ gene copies/g dry soil, with maximum values at either week 10 or 14 in the different treatments (Figure 3). To examine the general hydrocarbon degrading community during the remediation, qPCR was used to follow the number of gene copies of the *alkB* gene, a gene associated with alkane degradation [60] at different sampling times in all treatments (Figure 3). The abundance of the gene copies in all the treatments exhibited an inverted U-shaped pattern with time; this is consistent with another report [61]. Aziz et al. [12], which found that the hydrocarbon-utilising bacteria population generally increased with time initially, and later decreased with time in the amended treatments. In this current study, the abundance of this gene generally increased after the onset of remediation, peaking at either week 10 or 14 (Figure 3), which shows the utilisation of the substrate (alkane) by the bacteria. FTIR analyses further confirmed the utilisation of the alkane as the intensity of the peaks, which are associated with –CH_3_ and –CH_2_ groups in aliphatic compounds, decreased at week 10 compared to week 0 (Table S4). A decrease in the hydrocarbon utilising community occurred following the week 10 or 14 increases, likely due to the depletion of easily degradable hydrocarbon fractions, in this case alkane. Another possible explanation for this decrease could be that metabolites produced during the degradation of hydrocarbons have a negative effect on the metabolic activities of microorganisms [52].

## 4. Conclusions

This work examined the effects of immobilising *Ochrobacterium* sp. on biosolids-derived biochar (BIB) for the remediation of Australian soil contaminated with diesel. Findings from this study revealed that biochar enhanced hydrocarbon removal, especially at the early stage. Further, immobilisation of bacteria on biochar (BIB) led to a greater hydrocarbon removal than the application of biochar alone, which suggests the beneficial role of co-applying a hydrocarbon-degrading bacteria with biochar. However, the co-application of fertiliser slowed down the efficacy of BIB in hydrocarbon removal, which advocates for the need for caution and care when applying fertiliser. This study contributes to understanding the potential of biosolids-derived biochar in bacteria immobilisation and subsequent utilisation on the remediation of diesel-contaminated soil. Future studies should focus on how immobilisation studies involving biosolids-derived biochar could be further improved to achieve higher hydrocarbon removal, such as through biochar modification, immobilisation optimisation, etc. There is also a need for future studies to examine the effect of biosolids source on the efficacy of biochar with or without bacteria immobilization on the remediation of Australian soil contaminated with diesel.

## Figures and Tables

**Table 1:** Description of treatments used for the remediation of diesel-contaminated soil.

Treatments	Description	Identification Code
Control	No amendment	C
Bacteria	5 mL bacterial suspension (6.5 × 10^9^ CFU/mL)	B
Fertiliser *	2% *w*/*w* of fertiliser	F
Biochar	5% *w*/*w* of biochar	BC
Biochar with fertiliser *	5% *w*/*w* of biochar + 2% *w*/*w* of fertiliser	BCF
Bacteria immobilised biochar	5% *w*/*w* of biochar immobilised bacteria	BIB
Bacteria immobilised biochar co-application with fertiliser *	5% *w*/*w* of biochar immobilised bacteria + 2% *w*/*w* of fertiliser	BIBF

* Fertiliser: Yates thrive all-purpose soluble fertiliser—NPK 25:5:8.8. The fertiliser used is a quick release fertiliser.

## Data Availability

Data will be available on request.

## Supplementary Materials

The following supporting information can be downloaded at: https://www.mdpi.com/article/10.3390/toxics12040234/s1, Text S1: Polymerase chain reaction (PCR) and gel electrophoresis for primer; Table S1: Proximate analysis of pristine biochar and bacteria immobilised biochar [62,63]; Table S2: First-order kinetics equation, rate constant (k), half-life (t_1/2_) and R^2^, and of the different treatments; Table S3: Estimated time to achieve a concentration of 995–997 mg/kg, which is lower than the EPA Victoria fill material threshold (1000 mg/kg) in the different treatments; Table S4: The intensity of peaks associated with -CH_3_ and -CH_2_ in aliphatic compounds (peaks 2923 and 2853 cm^−1^) in the different treatments; Figure S1: Mechanism for hydrocarbon removal in soils amended with biochar immobilised microbe [17] (Copyright permission obtained).
