# Supplementary material for: Effect of the Application of Ochrobactrum sp.-Immobilised Biochar on the Remediation of Diesel-Contaminated Soil
Source: Toxics. 2024 Mar 22;12(4):234. doi: 10.3390/toxics12040234 (PMC11053889; doi:10.3390/toxics12040234)
Supplement: Supplementary file 1 [file toxics-12-00234-s001.zip › toxics-2851691-supplementary.pdf]

Supplementary File

Effect of the Application of *Ochrobactrum* sp.-Immobilised Biochar on the Remediation of Diesel-Contaminated Soil

Charles Chinyere Dike [1,2,*], Alka Rani Batra [1,2,3], Leadin S. Khudur [1,2], Kamrun Nahar [1,4] and Andrew S. Ball [1,2]

[1] ARC Training Centre for Transformation of Australia's Biosolids Centre, RMIT University, Bundoora,
Melbourne, VIC 3083, Australia; s3703623@student.rmit.edu.au (A.R.B.); leadin.khudur@rmit.edu.au (L.S.K.); s3855513@student.rmit.edu.au (K.N.); andy.ball@rmit.edu.au (A.S.B.)
[2] School of Science, RMIT University, Bundoora, Melbourne, VIC 3083, Australia
[3] Environment Protection Authority Victoria, Centre for Applied Sciences, Ernest Jones Drive,
Macleod, VIC 3085, Australia
[4] School of Engineering, RMIT University, Melbourne, VIC 3000, Australia
* Correspondence: charles.dike@student.rmit.edu.au

Materials and Methodology

Text S1: Polymerase chain reaction (PCR) and gel electrophoresis for primer

Polymerase Chain Reaction (PCR) was carried out in triplicate using the initial denaturation of 94 ^{o}C (4 min), followed by 35 cycles of 94 ^{o}C (30 s), 56 ^{o}C (30 s), and 72 ^{o}C (1 min) with a final extension of 15 min at 72 ^{o}C [1]. An aliquot (25 μL) was used for PCR, comprising of 2 x MIFI (12.5), 4 μL primer mix (10 μM each primer), 2 μL of template DNA of the bacteria and 8.5 μL PCR grade water. Following completion of the PCR run, a 5 μL PCR mixture was used to check for amplification on a 2% agarose gel in 1 x Tris-acetate-EDTA (TAE) buffer stained with SYBR safe DNA gel stain (Invitrogen, Massachusetts, US). Processing of the gel was carried out in the ChemiDoc (Bio-Rad, California, US) [2]

Results

Table S1: Proximate analysis of pristine biochar and bacteria immobilised biochar

Proximate analysis (wt% d.b)	Pristine biochar	Bacteria immobilised biochar
Moisture content (%)	0.42 ± 0.26	0.49 ± 0.30
Volatile matter (%)	3.15 ± 0.21	3.95 ± 0.21
Fixed carbon (%)	20.18 ± 5.26	25.26 ± 0.34
Ash content (%)	76.26 ± 4.79	70.30 ± 0.85

Values are mean of duplicate and the standard deviation of the mean.

Table S2: First-order kinetics equation, rate constant (k), half-life ($t_{1/2}$) and $R^{2,}$ and of the different treatments

Treatments	First order kinetic equation	k (day^{-1})	$t_{1/2}$ (days)	R^2
C	y=-0.0044x+11.096	0.0044	157	0.97
B	y=-0.0047x+11.108	0.0047	147	0.95
F	y=-0.0038x+11.176	0.0038	182	0.84
BC	y=-0.0049x+11.006	0.0049	141	0.96
BCF	y=-0.0046x+11.114	0.0046	151	0.95
BIB	y=-0.0053x+10.991	0.0053	131	0.99
BIBF	y=-0.0043x+11.092	0.0043	161	0.98

C: Control; B: Bacteria; F: 2% Fertiliser; BC: 5% w/w Biochar; BCF: 5% w/w Biochar + 2% Fertiliser; BIB: Bacteria immobilised biochar; BIBF: Bacteria immobilised biochar + 2% Fertiliser.

Table S3: Estimated time to achieve a concentration of 995 – 997 mg/kg, which is lower than the EPA Victoria fill material threshold (1,000 mg/kg) in the different treatments

	Time (weeks)	TPH conc at that time (mg/kg)
C	134	996
B	126	996
F	155	997
BC	120	996
BCF	128	997
BIB	111	996
BIBF	137	995

C: Control; B: Bacteria; F: 2% Fertiliser; BC: 5% w/w Biochar; BCF: 5% w/w Biochar + 2% Fertiliser; BIB: Bacteria immobilised biochar; BIBF: Bacteria immobilised biochar + 2% Fertiliser.

Table S4: The intensity of peaks associated with $-CH_3$ and $-CH_2$ in aliphatic compounds (peaks 2923 and 2853 cm^{-1}) in the different treatments

Wave number (cm^{-1})	Treatments	Intensity (Absorbance %)
2923	Week 0 contaminated soil	0.034 ± 0.0019
	C – Week 10	-0.014 ± 0.0066
	BC – Week 10	-0.0039 ± 0.01
	BIB – Week 10	-0.016 ± 0.0079
	C – Week 22	-0.018 ± 0.0035
	BC – Week 22	-0.017 ± 0.0052
	BIB – Week 22	-0.018 ± 0.0031
2853	Week 0 contaminated soil	0.020 ± 0.0021
	C – Week 10	-0.017 ± 0.0072
	BC – Week 10	-0.0078 ± 0.0089
	BIB – Week 10	-0.018 ± 0.0065
	C – Week 22	-0.0194 ± 0.0040
	BC – Week 22	-0.018 ± 0.0051
	BIB – Week 22	-0.019 ± 0.0030

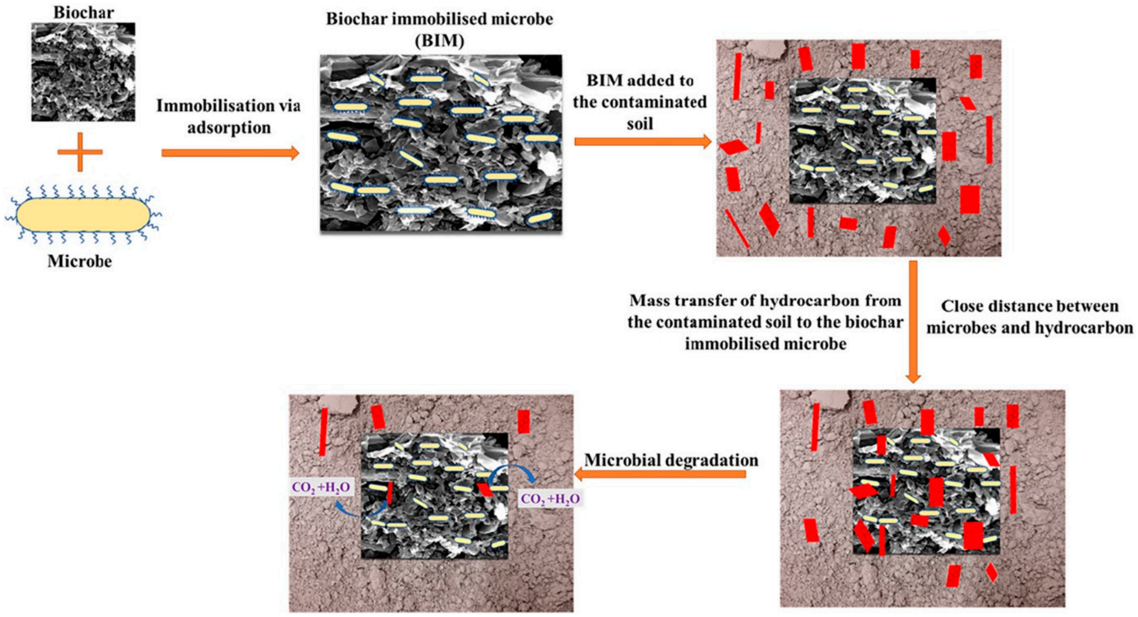

Figure S1: Mechanism for hydrocarbon removal in soils amended with biochar immobilised microbe [3] (Copyright permission obtained)

References

[1] Z. Liang, G. Li, B. Mai, H. Ma, T. An, Application of a novel gene encoding bromophenol dehalogenase from Ochrobactrum sp. T in TBBPA degradation, Chemosphere, 217 (2019) 507-515.

[2] G.K. Satyapal, S.K. Mishra, A. Srivastava, R.K. Ranjan, K. Prakash, R. Haque, N. Kumar, Possible bioremediation of arsenic toxicity by isolating indigenous bacteria from the middle Gangetic plain of Bihar, India, Biotechnology reports, 17 (2018) 117-125.

[3] C.C. Dike, I.G. Hakeem, A. Rani, A. Surapaneni, L. Khudur, K. Shah, A.S. Ball, The co-application of biochar with bioremediation for the removal of petroleum hydrocarbons from contaminated soil, Science of The Total Environment, 849 (2022) 157753.
